# Pulmonary Arteriovenous Malformation in a Rare Case of Hereditary Hemorrhagic Telangiectasia

**DOI:** 10.7759/cureus.32365

**Published:** 2022-12-09

**Authors:** Farhan Azad, Clive J Miranda, Gina M Sparacino

**Affiliations:** 1 Internal Medicine, University at Buffalo, Buffalo, USA; 2 Gastroenterology, University at Buffalo, Buffalo, USA

**Keywords:** epistaxis, telangiectasia, acvrl1, avm, hht

## Abstract

Hereditary hemorrhagic telangiectasia is a rare condition presenting with anemia requiring transfusion and nosebleeds often refractory to supportive therapy. We discuss a case of a male in his 60s with a history of epistaxis, anemia requiring transfusions, and acute on chronic worsening shortness of breath presenting for evaluation. He was diagnosed with hereditary hemorrhagic telangiectasia. In addition, he was found to have pulmonary arteriovenous malformations and nonbleeding gastric telangiectasias. The patient underwent coil embolization of pulmonary arteriovenous malformations with a resolution of his shortness of breath and his anemia improved with iron supplementation.

## Introduction

Hereditary hemorrhagic telangiectasia (HHT) or Rendu-Osler-Weber disease is a rare autosomal dominant disease with an incidence of 1:10,000 in North America [1]. It is manifested by the formation of arteriovenous malformation (AVM) with visceral involvement including the lungs, brain, gastrointestinal tract, and liver [2]. Small AVMs or telangiectasias are usually found in lips, tongue, and mucosal surfaces while large AVMs occur in visceral organs. AVMs allow blood to bypass capillaries between the artery and vein, making the vessels susceptible to rupture and predisposing the patient to anemia [3]. Patients can present with a multitude of symptoms depending on the organ involved. Shortness of breath, as seen in our patient, is common and it is difficult to determine the cause of it in HHT.

## Case presentation

A 60-year-old male from western Asia with a history of anemia requiring blood transfusions, lifelong epistaxis, and chronic shortness of breath presented with worsening shortness of breath. He had a history of nosebleeds that started when he was 20 years old, with prior cauterization, and now managed well with moisturizers. He always had shortness of breath attributed to his anemia which typically improved after transfusions. However, it got worse over the past week, especially on exertion. The last transfusion received was two months prior. He denied lightheadedness, cough, chest pain, and blood in stool or urine. Home medication included ferrous sulfate 325 mg every other day and he never received intravenous iron therapy. Family history was positive for multiple members with HHT confirmed with genetic testing. Social history was negative for tobacco or alcohol use. Vitals revealed a temperature of 37 degrees Celsius, blood pressure of 125/80 millimeters of mercury, heart rate of 80 beats per minute, and oxygen saturation of 96% in room air. On exam, he had small telangiectasias on the roof and diffuse small, scattered lesions elsewhere in the mouth. His notable laboratory investigations are shown in Table 1.

**Table 1:** Laboratory investigations MCV: mean corpuscular volume; MCH: mean corpuscular hemoglobin; MCHC: mean corpuscular hemoglobin concentration; LDH: lactate dehydrogenase; TSH: thyroid stimulating hormone; PT: prothrombin time; INR: international normalized ratio; PTT: partial thromboplastin time; TIBC: total iron-binding capacity

Laboratory investigations	Results	Reference Range
Peripheral Blood		
Hemoglobin	10.8 g/dL	13.5-17.5 g/dL
Hematocrit	35.1%	38.5-50 %
MCV	69.7 fL	80.0-100.0 fL
MCH	21.2 pg	27.0-33.0 pg
MCHC	29.6 g/dL	32.0-36.0 g/dL
Platelet count	306000/uL	140000-400000 uL
Reticulocyte count	1%	0.8-2.6%
Haptoglobin	119 mg/dL	32-363 mg/dL
LDH	576 IU/L	313-618 IU/L
TSH	3.728 uIU/mL	0.400-5.000 uIU/mL
Coagulation tests		
PT	13.3 seconds	9.4-12.5 seconds
INR	1.02	0.00-3.50
PTT	29.1 seconds	25.0-35.0 seconds
Initial iron panel		
Iron	46 ug/dL	38-169 ug/dL
TIBC	414 ug/dL	250-450 ug/dL
Iron saturation	11%	15-55 %
Ferritin	16.8 ng/mL	10-322 ng/mL
B12 and folate level		
B12	515 pg/mL	232-1245 pg/mL
Folate	21.2 ng/mL	>3.0 ng/mL

**Figure 1:**
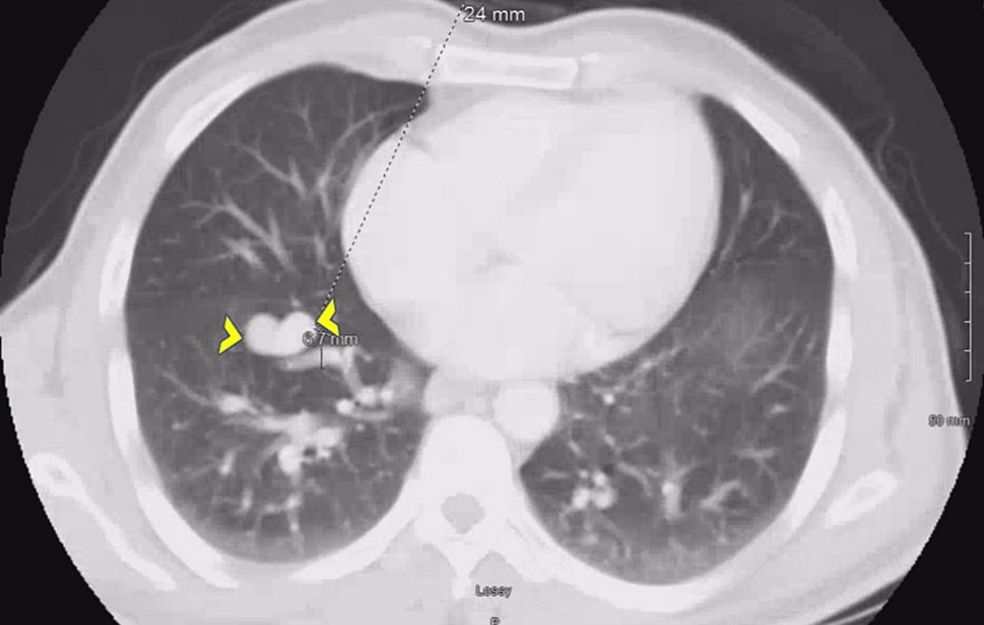
Computed tomography scan showing smooth lobular enhancing structure representing pulmonary arteriovenous malformation in the right lower lobe

Daily oral iron supplementation was initiated. Laboratory values three months later revealed hemoglobin of 14.7 g/dL (reference range: 13.5-17.5 g/dL), mean corpuscular volume of 90.2 fL (reference range: 80.0-100.0 fL), and ferritin of 30 ng/mL (reference range: 10-322 ng/mL). Computed tomography (CT) scan revealed right lower lobe pulmonary AVM (Figure 1).

Coil embolization was performed with the resolution of his shortness of breath. Endoscopy and colonoscopy showed nonbleeding telangiectasias and no interventions were done. Magnetic resonance imaging (MRI) of the head was negative for AVM. Subsequent visits continued to show stable hemoglobin levels on oral iron therapy. Genetic testing revealed that the patient is heterozygous for the p.R144X mutation in the ACVRL1 gene, consistent with the diagnosis of HHT.

## Discussion

The earliest sign of HHT is recurrent epistaxis occurring by the second decade of life, as seen in our patient. The AVMs can increase in size with age, causing gastrointestinal bleeding and anemia requiring transfusion, also seen in our patient’s history [4]. Genetic predisposition was evident as per the patient's extensive family history. It is a common monogenic disorder, with mutations in genes ENG and ACVRL1/ALK1 causing around 85% of the cases, the latter of which was seen in our patient [5]. The p.R144X mutation, located in exon 4 of the ACVRL1 gene, results from a C to T substitution at nucleotide position 430. This mutation was first described in a female patient with epistaxis, telangiectasias, and a family history of HHT [6]. It has also been described in several French HHT families [7]. Since premature stop codons are typically deleterious, this alteration is interpreted as a disease-causing mutation [8]. Diagnosis is made by the Curacao criteria which include epistaxis, telangiectasia, visceral lesions, and family history. HHT is suspected if two criteria are present, while three or more are definitive for HHT [9]. Our patient showed all four criteria confirming the diagnosis.

AVMs in the lungs, brain, and liver generally account for the fatal complications of HHT [10]. Pulmonary AVM (PAVM) is the most common and occurs in up to 30% of patients, clinically manifesting as right-to-left shunting and hypoxemia [11]. Since blood passing through PAVM bypasses the filtration system of capillaries, thrombi and other blood-borne pathogens are released into circulation, increasing susceptibility to cerebral embolic or septic events. Dyspnea is the most commonly reported symptom, although etiology can be difficult to distinguish due to the concurrent presence of iron deficiency anemia, high output heart failure, venous thromboembolism, and pulmonary hypertension [12]. Echocardiography is the modality of choice for screening while a CT scan is used for the diagnosis and characterization of PAVMs [13]. While most patients are asymptomatic, radiological evidence of PAVM regardless of size should be considered for catheter embolization, the treatment of choice, to reduce future ischemic events [14]. The patient promptly underwent PAVM embolization with the resolution of his symptoms.

HHT with PAVM and hypoxemia can have falsely elevated hemoglobin levels. Patients should have their iron status monitored closely regardless of their hemoglobin level. Guidelines recommend annual hemoglobin and serum iron monitoring, along with endoscopic evaluation when anemia is disproportionate to the amount of epistaxis [15]. The patient will continue to follow up at the leukemia clinic for monitoring of hemoglobin levels and symptoms pertinent to HHT.

## Conclusions

HHT is an autosomal dominant disorder that can present with multi-organ involvement, often requiring immediate intervention. Patients typically show disease manifestations as early as the second decade of life and often have an extensive family history of the diagnosis. Our case is a rare diagnosis of HHT with early management of pulmonary arteriovenous malformation, imperative in these patients. Our patient underwent coil embolization with immediate resolution of symptoms. With chronic anemia being a frequent complication of HHT, our patient will regularly follow up at the leukemia clinic for iron supplementation and blood transfusions as required.
